# Room Temperature NH_3_ Selective Gas Sensors Based on Double-Shell Hierarchical SnO_2_@polyaniline Composites

**DOI:** 10.3390/s24061824

**Published:** 2024-03-12

**Authors:** Yuan Qu, Haotian Zheng, Yuhua Lei, Ziwen Ding, Siqi Li, Song Liu, Wei Ji

**Affiliations:** 1 Key Laboratory of Forest Plant Ecology, College of Chemistry, Chemical Engineering and Resource Utilization, Northeast Forestry University, 26 Hexing Road, Harbin 150040, China; quyuan@nefu.edu.cn (Y.Q.); 2022213518@nefu.edu.cn (H.Z.); lyuh@nefu.edu.cn (Y.L.); 2023120911@nefu.edu.cn (Z.D.); carlosliusong@nefu.edu.cn (S.L.); 2 School of Chemistry, Dalian University of Technology, Dalian 116024, China

**Keywords:** NH_3_ sensor, double-shell, PANI@D-SnO_2_ composites, p-n heterojunction

## Abstract

Morphology and structure play a crucial role in influencing the performance of gas sensors. Hollow structures, in particular, not only increase the specific surface area of the material but also enhance the collision frequency of gases within the shell, and have been studied in depth in the field of gas sensing. Taking SnO_2_ as an illustrative example, a dual-shell structure SnO_2_ (D-SnO_2_) was prepared. D-SnO_2_@Polyaniline (PANI) (DSPx, x represents D-SnO_2_ molar content) composites were synthesized via the in situ oxidative polymerization method, and simultaneously deposited onto a polyethylene terephthalate (PET) substrate to fabricate an electrode-free, flexible sensor. The impact of the SnO_2_ content on the sensing performance of the DSPx-based sensor for NH_3_ detection at room temperature was discussed. The results showed that the response of a 20 mol% D-SnO_2_@PANI (DSP20) sensor to 100 ppm NH_3_ at room temperature is 37.92, which is 5.1 times higher than that of a pristine PANI sensor. Moreover, the DSP20 sensor demonstrated a rapid response and recovery rate at the concentration of 10 ppm NH_3_, with response and recovery times of 182 s and 86 s.

## 1. Introduction

Conductive polymers [1], owing to their mild synthesis conditions, high conductivity, and specific recognition capabilities towards target gases, have significant appeal in the field of gas sensors [2,3,4]. In particular, PANI is unique among the conductive polymers and is considered to be the most promising material for ammonia detection at room temperature owing to its specific deprotonation/protonation process for NH_3_ [5,6,7]. NH_3_, a colorless, unpleasant-smelling, highly toxic gas [8], can cause serious damage to immune systems and cell growth in humans with prolonged exposure [9], and the tolerance limit of humans drops drastically from 8 h to 15 min when the environmental concentration of NH_3_ rises from 25 ppm to 35 ppm [10,11]. Therefore, there is a big necessity for the capability to detect NH_3_ in room temperature environments. However, pristine PANI suffers from the drawbacks of low sensitivity and slow response in the field of NH_3_ sensing, necessitating modifications.

PANI-sensor-enhanced NH_3_-sensing performance primarily involves strategies such as the formation of p-n heterojunctions by combining PANI with an n-type metal oxide semiconductor (MOS) [12,13], the construction of hollow structures [14,15] and hierarchical structure gas-sensitive materials [16,17]. That is to say, the modulation of surface morphology is particularly essential to enhance the gas-sensitive performance of PANI, which mainly concerns the property enhancement brought by a large specific surface area, multiple active sites and a high porosity with microstructural advantages. For example, Jia et al. fabricated SnO_2_@PANI sensors based on core-shell nanotube structures via electrostatic spinning and plasma treatment, which exhibited an excellent and stable NH_3_ response and recovery properties at room temperature [18]. Li et al. successfully synthesized 20 mol% SnO_2_@Polyaniline (PASn20) sensitive materials with porous spherical structures on flexible substrates of PET by in situ polymerization, benefiting from the microstructure with a large specific surface area, the composites exhibited a 6.2-fold improvement towards room temperature response to NH_3_ [19]. Hong et al. successfully encapsulated PANI on In_2_O_3_ nanofibers with a hollow carbon coating by in situ polymerization and showed a response value of 18.2 for low concentrations of NH_3_ (1 ppm) at room temperature, which is 5.74 times higher than that of the pure PANI sensor [14]. Overall, PANI can exhibit different NH_3_-sensitive properties depending on the morphology of the MOS composite. Therefore, it is extremely meaningful to modulate the final growth and polymerization state of PANI according to the morphology of MOS for developing NH_3_-sensing materials with high sensitivity.

Hollow and hierarchically structured nanomaterials have gradually become a hotspot for the exploration of sensitive materials for gas sensors in recent years by virtue of their special structural advantages [20], such as a large specific surface area and high porosity [21], which are favorable for gas adsorption and diffusion. For example, Chen et al. adjusted the porosity of the prepared PANI composites with a core-shell layered structure by varying the parameters of coaxial electrostatic spinning to achieve gas detection with a high sensitivity and a low detection limit [22]. Gas-sensitive reaction processes primarily involve the activation of oxygen species and the adsorption–desorption processes of the target gas [23]. Specifically, this encompasses the diffusion of gas to the surface of the sensing material, collision and adsorption on the material surface, electron transfer between the adsorbed target gas and adsorbed oxygen, and the subsequent desorption processes [24,25,26,27]. The dual-shell hollow sphere structure possesses unique spatial effects, capable of altering the gas diffusion path and increasing the collision frequency, thereby enhancing the gas-sensing performance [28,29]. The dual-shell hollow sphere structure, characterized by its high specific surface area, large pore volume, and rapid reaction, has attracted attention in catalysis [30], drug delivery [31,32], and other fields [33]. The porous shell facilitates efficient gas transport; however, reports on multi-shell hollow structures for PANI-based sensors are extremely limited.

In this work, we successfully composite PANI with D-SnO_2_ by in situ chemical oxidative polymerization and prepared a series of room-temperature flexible NH_3_ sensors based on DSPx. Characterization by SEM, TEM, XRD, and FTIR proved the successful composite of the nanomaterials. Meanwhile, the NH_3_-sensing performance of the pre-pared DSPx sensors was evaluated at room temperature. The results showed that the DSP20-based sensor exhibited the best sensing performance for 100 ppm NH_3_ at room temperature, with a response value as high as 37.92, which is 5.1 times higher than that of pure PANI. The sensor also exhibited good response/recovery characteristics, repeatability, and long-term stability. Finally, the improved sensing performance is explained in terms of microstructure and p-n heterojunction.

## 2. Materials and Methods

### 2.1. Materials

Sucrose (C_12_H_22_O_11_, 99%, Aladdin, Shanghai, China), sodium hydroxide (NaOH, 95%, Macklin, Shanghai, China), crystalline tin tetrachloride (SnCl_4_·5H_2_O, 99.995%, Aladdin), potassium hydroxide (KOH, 90%, Rhawn, Shanghai, China), hydrochloric acid (HCl, 1M), ammonium persulfate (APS, AR, DaMao, Tianji, China), poly ethylene terephthalate, aniline (C_4_H_5_N), anhydrous ethanol (EtOH, AR, Chemical Reagent, Tianjin, China), PET (China Resources Chemical Materials Technology Co., Changzhou, China), and ammonia (NH_3_, 100 ppm and 1%, Liming Gas Co., Ltd., Harbin, China).

### 2.2. Fabrication of D-SnO_2_@PANI Composites

#### 2.2.1. Synthesis of D-SnO_2_ Microspheres

**Figure 1:**
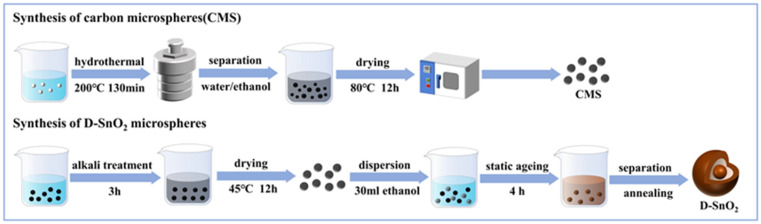
Schematic diagram of the preparation process of D-SnO_2_ microspheres.

As shown in Figure 1, a simple one-step hydrothermal method was used to synthesize uniformly shaped and sized carbon microspheres [29]. Firstly, carbon microsphere (CMS) templates with uniform size were obtained by dissolving sucrose in deionized water and utilizing the polymerization reaction of a sucrose emulsion under hydrothermal conditions. Then, D-SnO_2_ microspheres were prepared using CMS as sacrificial templates. The CMSs were dispersed ultrasonically in 100 mL of a 0.05 M NaOH solution and alkaline treated for 3 h at room temperature. Subsequently, the alkali-treated CMS templates and SnCl_4_·5H_2_O were sequentially added into 30 mL of anhydrous ethanol, ultrasonically dispersed for 30 min, and aged at room temperature for 4 h. Finally, D-SnO_2_ microspheres were obtained by alternately washing with deionized aqueous water and anhydrous ethanol, drying, and calcinating at 500 °C for 2 h, with a heating rate of 1 °C/min.

#### 2.2.2. Synthesis of D-SnO_2_@PANI Composite

**Figure 2:**
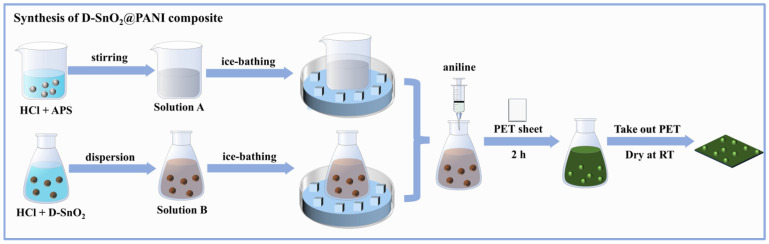
Schematic diagram of the preparation process of D-SnO_2_@PANI composite.

The DSPx binary composite was prepared by an in situ polymerization method [19]. As shown in Figure 2, firstly, 0.2 mmol of APS was added to 1 M HCl and stirred in an ice bath for 30 min to form Solution A. Then, a certain quantity of D-SnO_2_ powder was added to another portion of HCl and stirred in an ice bath to produce Solution B. Solution A was transferred to Solution B under ice-bath conditions, and then the fixed-size PET sheet (0.8 cm × 1.0 cm) was sequentially added to the solution and purified in aniline liquid by distillation, reacting for 2 h away from light. Finally, the PET sheets containing the DSPx-sensitive material were removed and left to be dried naturally at room temperature within 24 h so as to obtain gas-sensitive elements that can be used for gas-sensing tests. In this experiment, we modulated the molar ratios of the added D-SnO_2_ and aniline ($n{\;}_{D-SnO_{2}}$: $n_{aniline}\;$× 100% = 0%, 10%, 20%, 30%, and 60%, respectively) to prepare a series of DSPx-sensitive elements and explored the effect of different compounding amounts on gas-sensing performance.

### 2.3. Material Characterization

The crystallographic properties of DSPx were characterized by a Rigaku D/Max-2550 V X-ray powder diffractometer (XRD) irradiated with Cu-Kα rays (λ = 0.15406 nm) in the range of 5–80° at a scanning speed of 10° min^−1^. Field emission scanning electron microscopy (FESEM, JEOL JSM-7500F) and transmission electron microscopy (TEM, JEOL JEM-2100F) were used to reveal the micro-morphology and compositional structure of the materials at 5 kV. Fourier transform infrared spectroscopy (FT-IR, Spectrum 400) was used to characterize the chemical structure of the materials. The Brunauer–Emmett–Teller (BET) theory and the Barrett–Joyner–Halenda (BJH) model were used to measure the surface area and pore-size distribution of the materials, respectively.

### 2.4. NH_3_-Sensing Measurement

**Figure 3:**
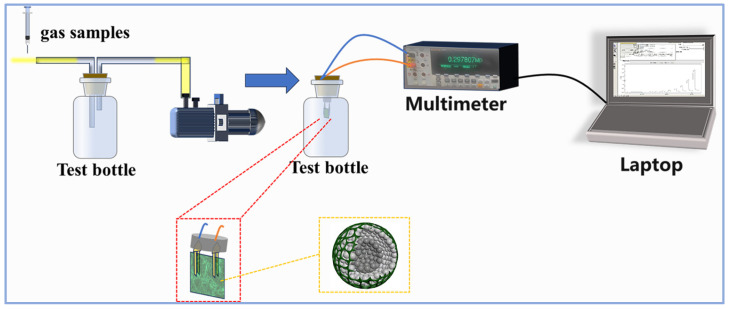
The diagram of the NH_3_ measurement device.

The gas-sensing measurement platform was designed by exposing the sensing device to different concentrations of NH_3_ at room temperature and recording the change in material resistance with a Fluke meter. The measurement device is shown in Figure 3. Firstly, we prepared two 1 L stoppered glass bottles and used a vacuum pump with a three-way switch to remove all the gases from the bottles leading to a negative pressure inside the bottles. Then, the knob of the three-way switch was adjusted to fill only one bottle with fresh air, and the other bottle with a mixture of NH_3_ and fresh air. The filling amount of NH_3_ was strictly controlled using a microfeeder. Next, the gas-sensitive test element fitted with a PET sheet was placed into the glass bottle filled with fresh air, the other end of the element was connected to a Fluke meter via a connecting cable, and the change in resistance of the sensitive materials was recorded in real time by an electronic computer. Once the resistance of the test element had stabilized, it was quickly transferred to the glass bottle containing test gases, at which point a large change in resistance occurred. When the resistance of the element stabilized in the test bottle, it was returned to the air bottle for the next test. We define the response of the sensor as S = R_g_/R_a_, where R_g_ is the resistance in the presence of the test gas and R_a_ is the resistance in air. The response and recovery time are defined as the time taken to realize a 90% change in total resistance for the sensor before and after exposure to the target gas.

## 3. Results and Discussion

### 3.1. Structural and Morphological Characteristics

**Figure 4:**
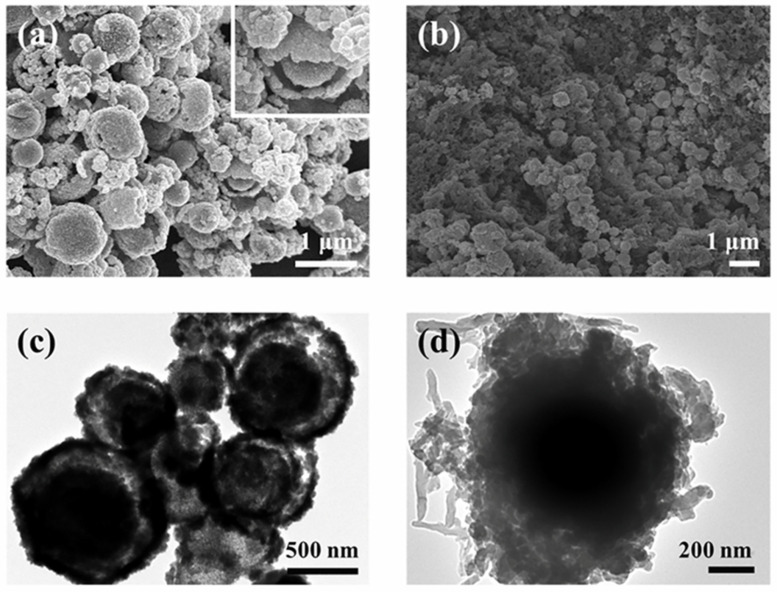
SEM images of (**a**) D-SnO_2_; (**b**) DSP20; TEM images of (**c**) D-SnO_2_; (**d**) DSP20.

The morphology and microstructure of the prepared D-SnO_2_ and DSP20 were observed by SEM and TEM as shown in Figure 4. It can be seen that the prepared D-SnO_2_ exhibits a hierarchical double-shell spherical structure composed of numerous small particles and the diameter is approximately 550 nm (Figure 4a). The emergence of permeable apertures conducive to gas transmission became evident on the surface of the hierarchical microspheres, and was attributed to the sparse arrangement of the diminutive SnO_2_ particles. In addition, the TEM image in Figure 4c further confirms the microstructure of the double-layer hierarchy of spheres. In Figure 4b,d, the synthesized DSP20 consistently retained the spherical morphology characteristic of D-SnO_2_. Significantly, polyaniline was intricately grown in situ on the exterior of D-SnO_2_, creating a conductive network structure. The effective integration of PANI with D-SnO_2_ is anticipated to facilitate an expedited electron transfer rate, thereby augmenting the gas-sensitive properties of the materials.

**Figure 5:**
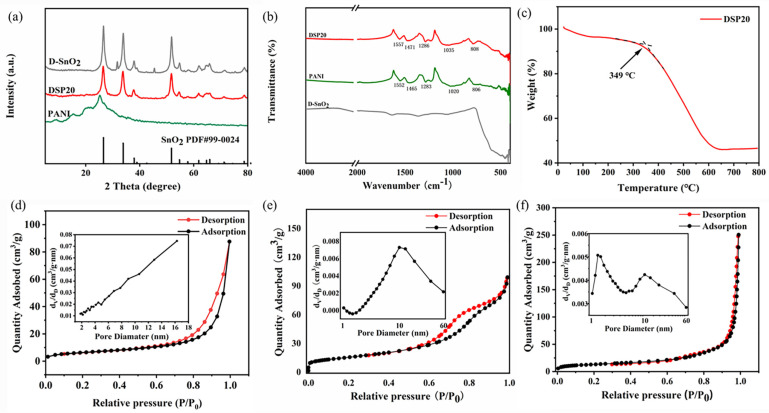
(**a**) X−ray diffractograms of D−SnO_2_, PANI, and in situ−polymerized DSP20 samples; (**b**) FTIR spectra of D−SnO_2_, PANI, and DSP20 samples; (**c**) the thermo−gravimetric analysis curves of DSP20 composites; nitrogen adsorption−desorption isotherms and pore-size distributions of (**d**) PANI, (**e**) D−SnO_2_, and (**f**) DSP20.

The XRD patterns of the prepared samples were scanned for peaks in the test range of 5–80°, as shown in Figure 5a. In the XRD spectrum of PANI, the appearance of broad diffraction peaks between 10 and 30° can be attributed to the parallel and perpendicular periodicity of the polyaniline chains [34]. The diffraction peaks of D-SnO_2_ were observed at 2θ = 26.08°, 33.78°, 38.04°, 51.72°, 54.63°, 61.81°, 65.84°, and 71.22°, which correspond to the (110), (101), (200), (211), (220), (310), (301), and (202) crystal planes, respectively. All these peaks can correspond to the standard card of SnO_2_ (PDF#99-0024). The XRD pattern of DSP20 was similar to that of D-SnO_2_, indicating that there was no change in the crystalline state of D-SnO_2_ complexed with polyaniline.

The FTIR spectra of PANI, D-SnO_2_, and DSP20 are shown in Figure 5b. For the PANI spectrum, the main characteristic absorption peaks are located at 1552, 1465, 1283, 1020, and 806 cm^−1^ [35,36,37]. In this case, the absorption peak centered at 1552 cm^−1^ was attributed to the C=C stretching vibration of the quinone ring (N=Q=N) and the absorption peak centered at 1465 cm^−1^ was attributed to the absorption vibration of the benzene structure (N-B-N). The peak located at 1283 cm^−1^ is attributed to the C-N stretching mode of the benzene unit. The peaks at 1020 and 806 cm^−1^ indicate the in-plane and out-of-plane C-H bending vibrations of the benzene ring, respectively. In the spectrum of D-SnO_2_, the two weak peaks shown in the region of 500–750 cm^−1^ are assigned to the vibrational bands of the Sn-O bonds [38]. The FTIR spectra of DSP20 contained the principal characteristic peaks of PANI. Moreover, under the influence of SnO_2_, these peaks exhibit a high wavenumber shift, specifically at 1557, 1471, 1286, 1035, and 808 cm^−1^.

The DSP20 composites were characterized by thermogravimetric analysis and the results are shown in Figure 5c. The DSP20 composites exhibit a small amount of mass loss until the temperature reaches 100 °C, which is caused by the release of water molecules absorbed on the surface of the materials. The weight loss between 100 °C and 349 °C can be attributed to the elimination of dopant HCl in the acid-doped PANI. The major weight loss between 349–648 °C could be ascribed to the decomposition of PANI [39]. The weight of DSP20 composite hardly changed within the temperature range of 648–800 °C, remaining at 46.6%.

N_2_ adsorption–desorption results of PANI, D-SnO_2_, and DSP20-sensitive materials are shown in Figure 5d–f. Among them, the specific surface area of the pure PANI is 22.771 m^2^/g (Figure 5d). BET was used to calculate the specific surface area of the DSP20-sensing materials (46.619 m^2^/g), and a more concentrated pore size distribution was found at 2 nm and 10 nm (Figure 5f). Compared with pure PANI, the specific surface area of DSP20-sensing materials increased remarkably. This increase can lead to the exposure of multiple active sites and the enhancement of gas adsorption and desorption, thus improving the sensing performance [40]. Considering that the aniline monomer is polymerized on the surface of D-SnO_2_, it is inevitable that the specific surface area of the DSP20 composites would be slightly smaller than that of D-SnO_2_ (55.951 m^2^/g). Notably, the successful composite of PANI and D-SnO_2_ forms electrically conductive networks that can effectively accelerate the electron transport rate and enhance the gas-sensitive performance.

### 3.2. Gas-Sensing Properties

**Figure 6:**
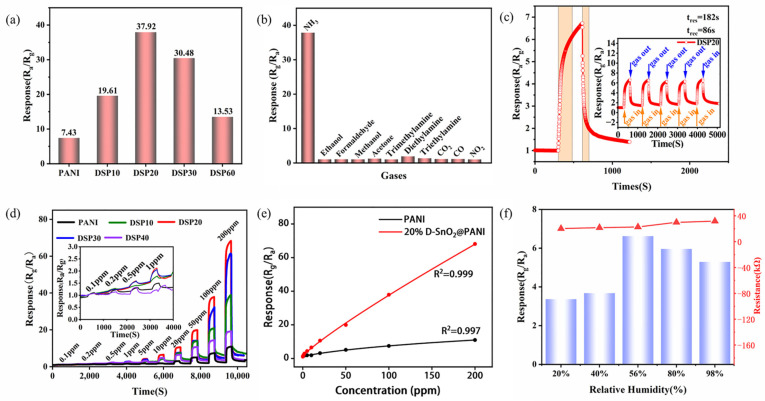
(**a**) Effect of different molar addition percentages of D-SnO_2_ and PANI on the sensing performance of sensors at room temperature and an ammonia concentration of 100 ppm; (**b**) the selectivity of DSP20-based sensors towards different gases with 100 ppm concentration at room temperature; (**c**) transient curves of the response and recovery of the DSP20-based sensor to 10 ppm concentration of ammonia at room temperature, and transient curves of the recovery of the response of the DSP20 sensor to 10 ppm NH_3_ five consecutive times at room temperature in the inset; (**d**) recovery curves of the dynamic response of the PANI- and DSP-based sensors to 0.1–200 ppm NH_3_ at room temperature, and the inset shows a localized zoomed-in plot of the dynamic response of all the sensors at concentrations of 0.1–1 ppm NH_3_; (**e**) nonlinear fitting curves of the response of the PANI- and DSP20-based sensors to 0.1–200 ppm NH_3_ at room temperature; (**f**) the response and fundamental resistance plots of the DSP20-based sensor under 10 ppm NH_3_ at different relative humidities.

The NH_3_-sensing performances of the pure PANI and DSPx-based sensors were evaluated at room temperature with a relative humidity of about 60%. As observed in Figure 6a, the composite with D-SnO_2_ significantly influences the NH_3_-sensing capabilities. The response of the pure PANI-based sensor at a concentration of 100 ppm NH_3_ is only 7.43, with an increase in the content of D-SnO_2_, the response exhibits a distinct “volcanic” trend. Specifically, when the molar composite ratio of D-SnO_2_ to aniline is below 20%, the relative diffusion of electrons and holes between them forms a number of p-n heterojunctions conducive to enhanced NH_3_ sensing, which results in the gradually enhanced sensing characteristics exhibited by PANI, DSP10, and DSP20-based sensors. However, as the molar composite ratio of the two continues increasing from 20% to 60%, the proportion of D-SnO_2_ in the composite will increase dramatically and compete for NH_3_ adsorption [39], resulting in a decrease in the amount of NH_3_ that should have been adsorbed by the gas-sensitive active sites, leading to a decrease in the performance of the DSP30 and DSP60-based sensors. Particularly noteworthy is the performance of the sensor based on DSP20, demonstrating a prominent maximum response of 37.92 to 100 ppm NH_3_, which is 5.1 times higher than that of the pure PANI sensor.

The selectivity of the DSP20-based sensor was evaluated at room temperature over various gases at a concentration of 100 ppm, and the results are shown in Figure 6b. The response of the DSP20-based sensor for NH_3_, ethanol, formaldehyde, methanol, acetone, trimethylamine, diethylamine, triethylamine, CO_2_, CO, and NO_2_ under the same conditions are 37.92, 1.04, 1.05, 1.03, 1.24, 1.01, 1.91, 1.35, 1.10, 1.14, and 1.01 respectively, which indicates excellent sensing properties for NH_3_. In Figure 6c, the dynamic response and recovery characteristics of the DSP20-based sensor are demonstrated at a concentration of 10 ppm NH_3_. According to the definition of t_res_ and t_rec_, the response and recovery time of the prepared DSP20 sensor for 10 ppm NH_3_ are 182 s and 86 s, respectively. Furthermore, as shown in the inset of Figure 6c, the sensor also exhibits excellent repeatability, as it is capable of exhibiting the same response and recovery characteristics over five consecutive test cycles.

Figure 6d describes the dynamic response of the sensors prepared using pure PANI and DSPx at different concentrations (0.1–200 ppm) of NH_3_ at room temperature. With the NH_3_ concentration increasing, the response of all the sensors showed an increasing trend. The gas-sensing performances of the PANI and DSPx-based sensors were varied. Of these, the DSP20-based sensors showed the most remarkable improvement and the response to NH_3_ concentrations of 0.1 ppm, 0.2 ppm, 0.5 ppm, 1 ppm, 5 ppm, 10 ppm, 20 ppm, 50 ppm, 100 ppm, and 200 ppm were 1.07, 1.25, 1.59, 2.10, 2.89, 4.35, 6.13, 6.53, 10.61, 19.96, 37.92, and 68.04, respectively. The fitting curves depicting the variation with NH_3_ concentration for pure PANI and DSP20-based sensors are illustrated in Figure 6e. The fitting equation based on the DSP20 sensor is given as y = 1.06602 + 0.63772 × |x − 0.1|^0.87739, and adheres to the typical model for composite gas sensing, and the corresponding R^2^ value is 0.999. According to this equation, the calculated theoretical detection limit is 268 ppb (response value of 1.2).

We also investigated the sensing performance of DSP20-based sensors for 10 pm NH_3_ at different humidity levels, and the results are shown in Figure 6f. As the relative humidity (RH) increases from 20% to 56%, the response of the sensor gradually increased and reached a maximum value of 6.62 at 56% RH. This phenomenon can be attributed to the in-doping effect of water on the acidified PANI. After that, with a further increase in relative humidity, the presence of more water molecules occupies the active sites which are meant to be sensitively reactive to NH_3_ gas [41], thus leading to the degradation of the sensor performance.

Additionally, the long-term stability of the sensor based on DSP20 was evaluated for its response to 10 ppm NH_3_ at room temperature over a continuous period of 17 days. As shown in Figure S1, the response of the sensor drops to ~50% of the initial value on the 3rd day and then stabilizes. The factors responsible for this phenomenon are the aging of the PET film and the disappearance of the unstable adsorption sites.

The comparison of the sensing performance of DSP20-based sensors with previously reported devices is provided in Table 1. Notably, the DSP20-based sensors proposed in this research exhibit superior performance in terms of both sensitivity and detection limit, and have favorable application prospects.

### 3.3. Gas-Sensing Mechanism

Polyaniline undergoes acid doping under acidic conditions, forming polarons and transforming into the conductive emeraldine-salt state of polyaniline. Specifically, the acid produces protons and enters the chain of PANI to combine with the N atom on the imine group (=N-), causing the quinone ring in the structure of PANI to be reduced to a benzene ring and forming a polaron, which makes the less-than-conducting eigenstates of polyaniline electrically conductive and results in an emerald-green imine-salt state. This doping process is reversible; when exposed to an NH_3_ environment, a de-doping reaction occurs. Hence, this distinctive chemical reaction imparts a specific detection functionality to polyaniline for NH_3_. The reversible chemisorption process occurring between PANI and NH_3_ molecules is shown in Equation (1). When the sensor was exposed to NH_3_, NH_3_ captures H^+^ from the -NH- groups, leading to a reduction in the polaron density along the main chain of polyaniline, rendering the PANI in a highly resistive state. The opposite process would occur when the sensor was placed back in air [16].

(1) $$PANI-H^{+}+NH_{3}↔PANI+NH_{4}^{+}$$

**Figure 7:**
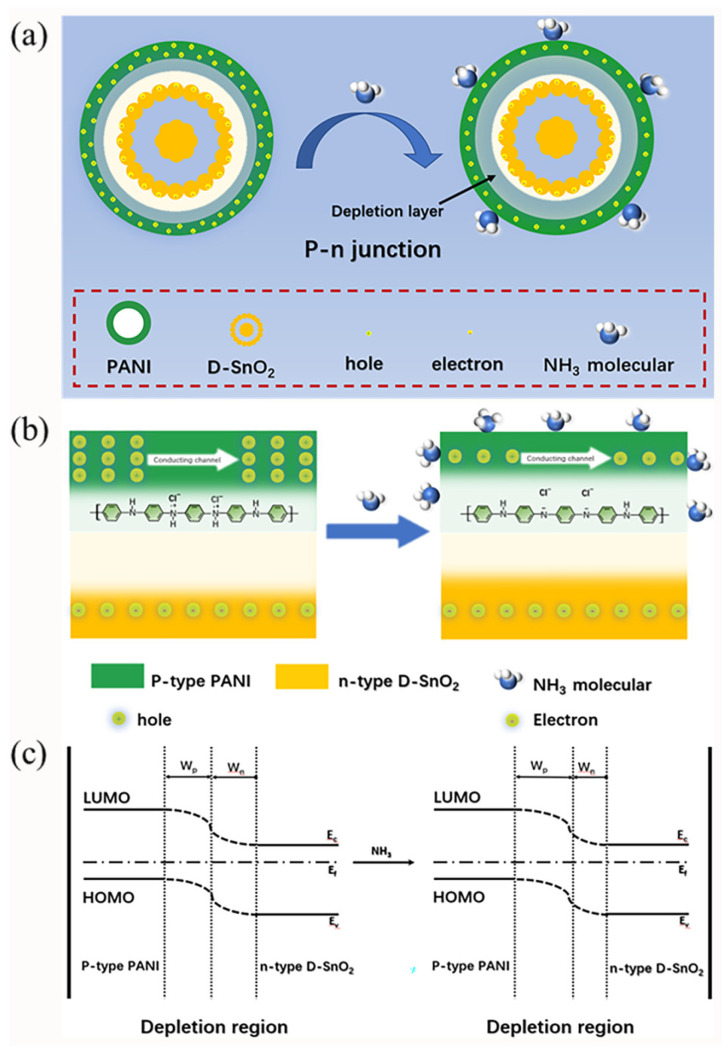
Sensing mechanism of DSP20 in air and NH_3_: (**a**) formulation process of p-n heterojunction; (**b**) local magnification of heterojunction formation; and (**c**) schema of energy band structure.

The dramatic improvement in the sensing performance of the DSP20 compound can be mainly attributed to the following two aspects: firstly, the composite exhibits a dual-shell microstructure, featuring a substantial specific surface area and permeable pores, facilitating enhanced gas diffusion and adsorption [37]. Additionally, when the n-type D-SnO_2_ surface undergoes in situ polymerization with p-type proton-doped polyaniline (PANI-H^+^), the holes in PANI-H^+^ and the electrons in D-SnO_2_ mutually diffuse. This results in the formation of a p-n heterojunction at the interface, as illustrated in Figure 7. When the sensor is exposed to air, driven by the energy level difference between PANI-H^+^ and D-SnO_2_, the holes in PANI-H^+^ and the electrons in D-SnO_2_ diffuse in opposite directions, forming a narrow depletion layer. This leads to a re-balancing of the Fermi level, resulting in a lower-resistance state for the sensing material. Upon exposure to NH_3_, NH_3_ captures the H^+^ in PANI-H^+^, leading to a further increase in the width of the depletion layer, causing a sharp rise in material resistance. Hence, the signal amplification of the p-n heterojunction in this reaction significantly enhances the sensing performance of DSP20.

## 4. Conclusions

In summary, a double-shell SnO_2_ with a permeable pore structure via a sacrificial template method were prepared, and through the in situ polymerization of PANI simultaneously deposited onto PET substrates to fabricate room-temperature NH_3_ sensors, namely DSPx sensors. The results show that DSPx-based sensors enhanced the NH_3_ sensing performance to varying degrees. In particular, the DSP20-based sensor exhibits excellent gas-sensing performance, with a response value of 37.92 to 100 ppm NH_3_ and excellent selectivity. In addition, it has good response recovery characteristics, repeatability and acceptable long-term stability. This improved performance is attributed to its special microstructure and the p-n heterojunction formed between the D-SnO_2_ surface and PANI. Therefore, this DSP-based sensitive material is expected to become an ideal material for the highly selective detection of NH_3_ at room temperature.

## Figures and Tables

**Table 1:** Comparison of the sensing properties of DSP20-based sensor in this study with previously reported devices.

Materials	Temperature	Target Gas	Sensitivity	Detection Limit	References
PANI	RT	NH_3_	19.46–100 ppm	2 ppb	[42]
PANI	RT	NH_3_	5.4–40 ppm	0.1 ppm	[8]
Ag-ZnO/PANI	RT	NH_3_	50%–100 ppm	5 ppm	[43]
rGO/PANI/TPU	RT	NH_3_	1.08–100 ppm	5 ppm	[44]
SnO_2_@PANI	RT	NH_3_	1.018–100 ppm	0.4 ppm	[37]
PANI/WO_3_	RT	NH_3_	1.25–3 ppm	192 ppb	[45]
PANI/MoS_2_/SnO_2_	RT	NH_3_	10.9–100 ppm	200 ppb	[46]
DSP20	RT	NH_3_	37.92–100 ppm	268 ppb	This work

## Data Availability

Dataset available on request from the authors.

## Supplementary Materials

The following supporting information can be downloaded at: https://www.mdpi.com/article/10.3390/s24061824/s1, Figure S1: Long-term stability curve of the DSP20-based sensor response to 10 ppm NH3 at room temperature.
