# Supplementary material for: Room Temperature NH3 Selective Gas Sensors Based on Double-Shell Hierarchical SnO2@polyaniline Composites
Source: Sensors (Basel). 2024 Mar 12;24(6):1824. doi: 10.3390/s24061824 (PMC10975220; doi:10.3390/s24061824)
Supplement: Supplementary file 1 [file sensors-24-01824-s001.zip › sensors-2878523-supplementary.pdf]

sensors

MDPI

Room Temperature NH_3 Selective Gas Sensors Based on Double-Shell Hierarchical SnO_2@polyaniline Composites

Yuan Qu [1,†], Haotian Zheng [1,†], Yuhua Lei [1], Ziwen Ding [1], Siqi Li [1,*], Song Liu [1] and Wei Ji [1,2,*]

[1] Key Laboratory of Forest Plant Ecology, College of Chemistry, Chemical Engineering and Resource Utilization, Northeast Forestry University, 26 Hexing Road, Harbin 150040, China; quyuan@nefu.edu.cn (Y.Q.); 2022213518@nefu.edu.cn (H.Z.); lyuh@nefu.edu.cn (Y.L.); 2023120911@nefu.edu.cn (Z.D.); carlosliusong@nefu.edu.cn (S.L.)

[2] School of Chemistry, Dalian University of Technology, Dalian 116024, China

* Correspondence: lisiqi@nefu.edu.cn (S.L.); jiwei@dlut.edu.cn (W.J.)

† These authors contributed equally to this work.

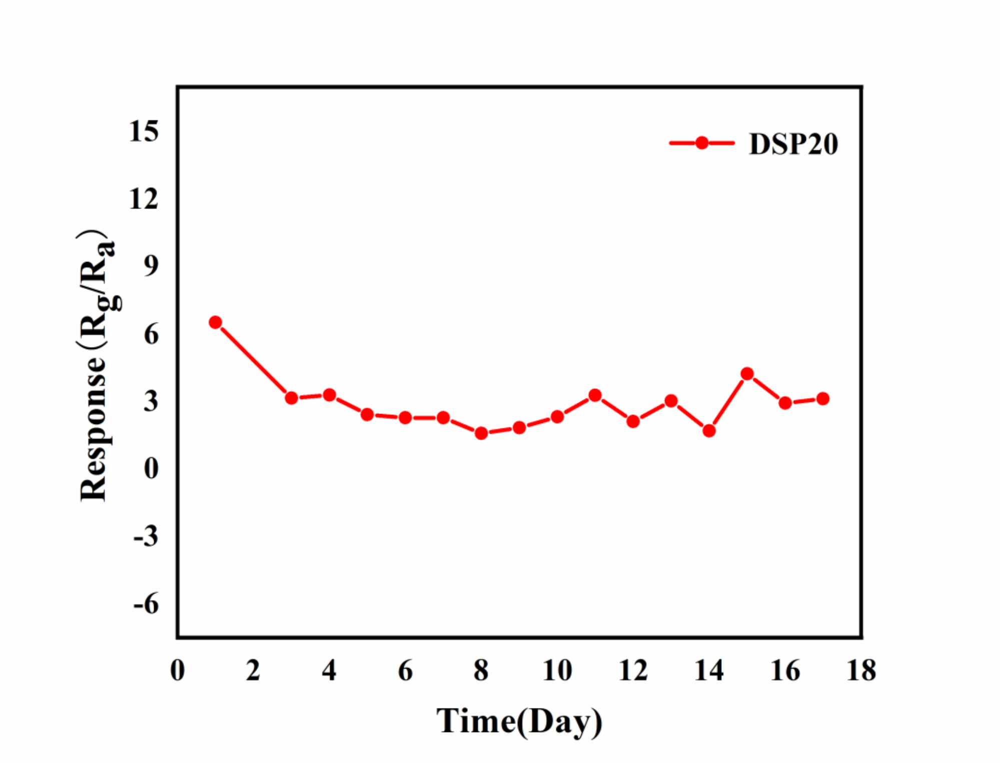

Figure S1. Long-term stability curve of the DSP20-based sensor response to 10 ppm NH_3 at room temperature.
